# Siderotic glaucoma without detectable intraocular foreign body in a pseudophakic eye: a case report

**DOI:** 10.1186/s12886-020-01691-8

**Published:** 2020-10-19

**Authors:** Yang Huang, Zi Ye, Zhaohui Li

**Affiliations:** Department of Ophthalmology, The Chinese People’s Liberation Army General Hospital, No.28 Fuxing Road Haidian District, Beijing, China

**Keywords:** Ocular siderosis, Glaucoma, Intraocular foreign body, Histopathology, Case report

## Abstract

**Background:**

Ocular siderosis is induced by a retained intraocular foreign body (IOFB) containing iron and can present as siderotic glaucoma. We report a rare case of histopathologically proven siderotic glaucoma in a middle-aged blacksmith with a preceding history of ocular trauma but no radiologically detectable IOFB.

**Case presentation:**

A 42-year-old blacksmith presented with an elevation of intraocular pressure (IOP) in left eye showing iris heterochromia and brownish deposits throughout the trabecular meshwork (TM). Preoperative ophthalmic examination did not reveal any retained IOFBs. Electroretinography showed the classic changes of retinal degeneration in ocular siderosis. Histopathologic staining of the TM verified the presence of iron deposits.

**Conclusion:**

This case underlines the importance of the close monitoring of patients with a history of ocular trauma and highlights the necessity of electroretinography, histopathologic study, and detailed ophthalmic examination in the diagnosis of siderotic glaucoma, even if there is no definite radiologically detectable IOFB.

## Background

The retained intraocular foreign body (IOFB) containing iron in the eye can induce a sight-threatening ocular siderosis, which may occur 18 days to 8 years after the ocular injury [1]. The clinical manifestations of ocular siderosis include decreased visual acuity, iris heterochromia, anisocoria with dilated pupil, brownish rust-like deposition on the anterior lens capsule, cataract formation and retinal pigment changes or degeneration [2–4]. Retinal damage caused by the deposition of iron released from the IOFB may result in the disorganization of the retina with loss of cholinergic amacrine cells and photoreceptor outer segments, which often presents with characteristic changes on electroretinography (ERG) [5, 6]. In addition, ocular siderosis also leads to siderotic glaucoma [7, 8].

In general, siderotic glaucoma is easy to diagnose in eyes with a retained IOFB containing iron. Siderotic glaucoma is presented as an elevation of intraocular pressure (IOP), glaucomatous damage and distinct amplitude loss in the scotopic, photopic, and Flicker responses measured by ERG [5, 9, 10]. It is postulated that a ferrous IOFB may undergo dissociation leading to the deposition of iron in the trabecular meshwork (TM), thus decreasing the outflow facility [11]. Nevertheless, for those without definite, radiologically detectable IOFB, misdiagnosis may occur. We report a rare case of histopathologically confirmed siderotic glaucoma in a middle-aged man with no signs of retained IOFB.

## Case presentation

A 42-year-old blacksmith presented with progressive blurred vision, intermittent eye pain, and a change in the colour of his left eye that he had noticed 6 months ago. On detailed questioning, he told us that over 2 years ago, his left eye was hit by an iron stick when performing steel maintenance. At the time, he was assessed in a local hospital, but he was reassured that he only had an eye contusion. According to the medical records, the visual acuity was 10/20 with mild foreign body sensation and conjunctival congestion. No wounds or foreign bodies were observed in the conjunctiva and cornea. In addition, there was no subconjunctival haemorrhage. The visual acuity recovered to 20/20 2 days after topical levofloxacin administration (0.5%, qid). Four months later, however, his visual acuity in the left eye deteriorated to 1/20, and he was diagnosed with “traumatic cataract”. During the examination, pigmented keratic precipitates (KPs), iris heterochromia and mydriasis were not present. Ultrasound B-scan and ultrasonic biomicroscopy (UBM) revealed no IOFB. Computed tomography (CT) was not performed. Then, he underwent phacoemulsification and post-chamber intraocular lens (PC-IOL) implantation at a local hospital, and postoperative visual acuity reverted to 20/20.

**Fig. 1:**
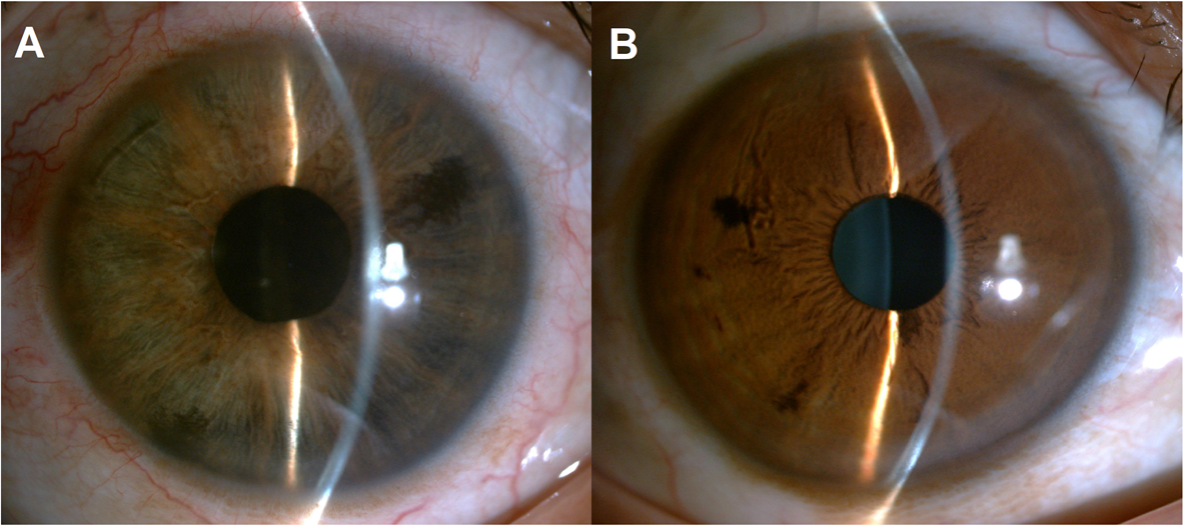
Slit-lamp images showing iris heterochromia. **a**, Iris depigmentation and mild mydriasis were observed in the left eye. **b**, Normal iris in the right eye

**Fig. 2:**
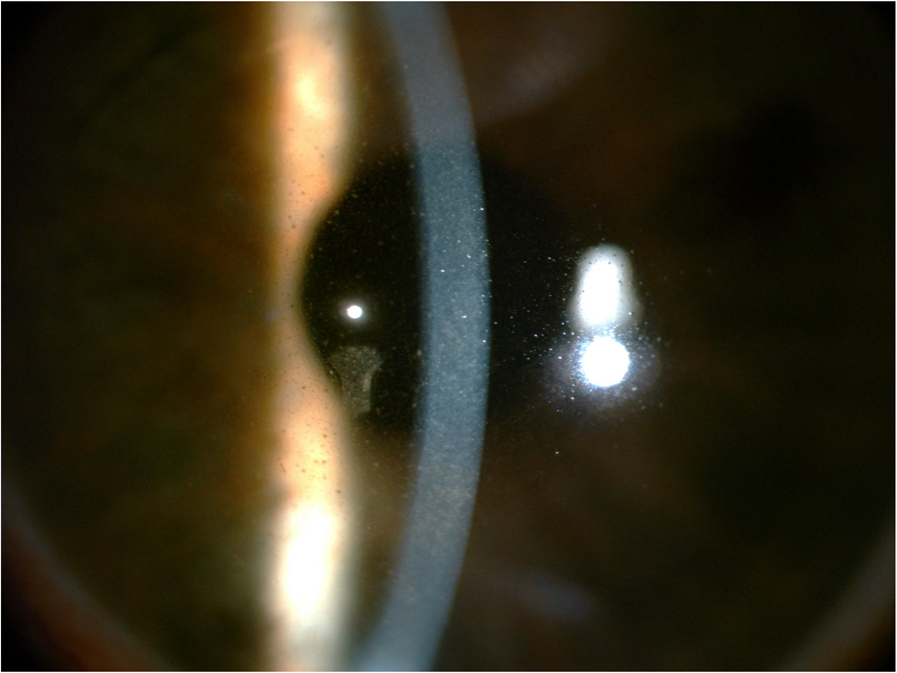
Direct focal illumination image focusing on the posterior surface of the cornea of the left eye showing mild corneal edema and diffuse pigmented keratic precipitates without visible wounds or scars

**Fig. 3:**
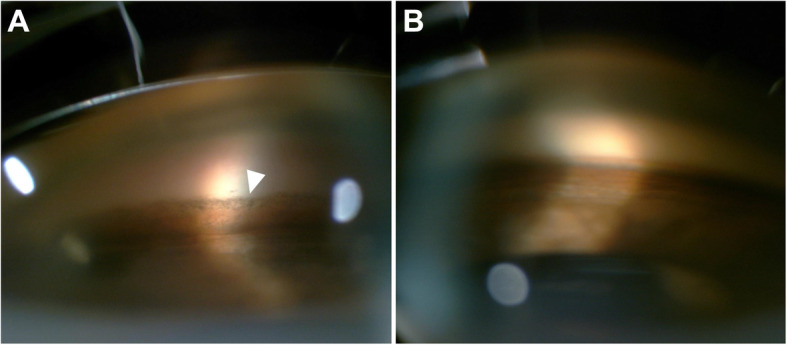
Gonioscopic images of both eyes. **a**, An open chamber angle with a line of brownish deposits was observed on the anterior trabecular meshwork in the left eye (white arrowhead). **b**, The right eye revealed an open chamber angle with scattered pigmentation on the anterior trabecular meshwork

**Fig. 4:**
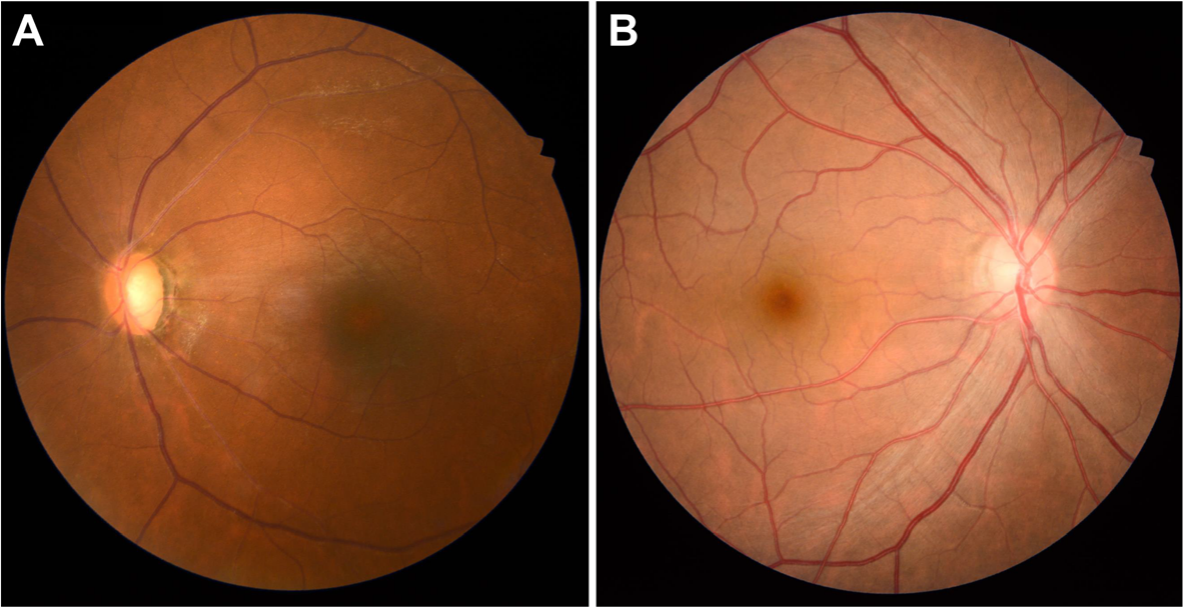
Posterior segment fundus images of the left eye (**a**) and the fellow eye (**b**)

**Fig. 5:**
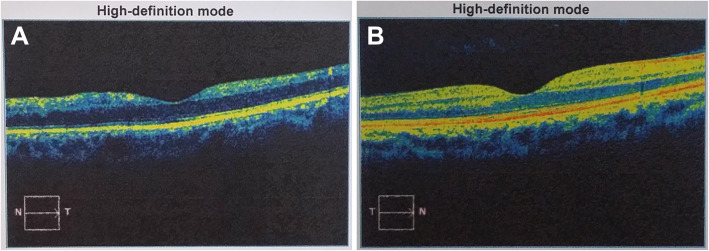
Central macular OCT scan of the left eye (**a**) and the fellow eye (**b**)

**Fig. 6:**
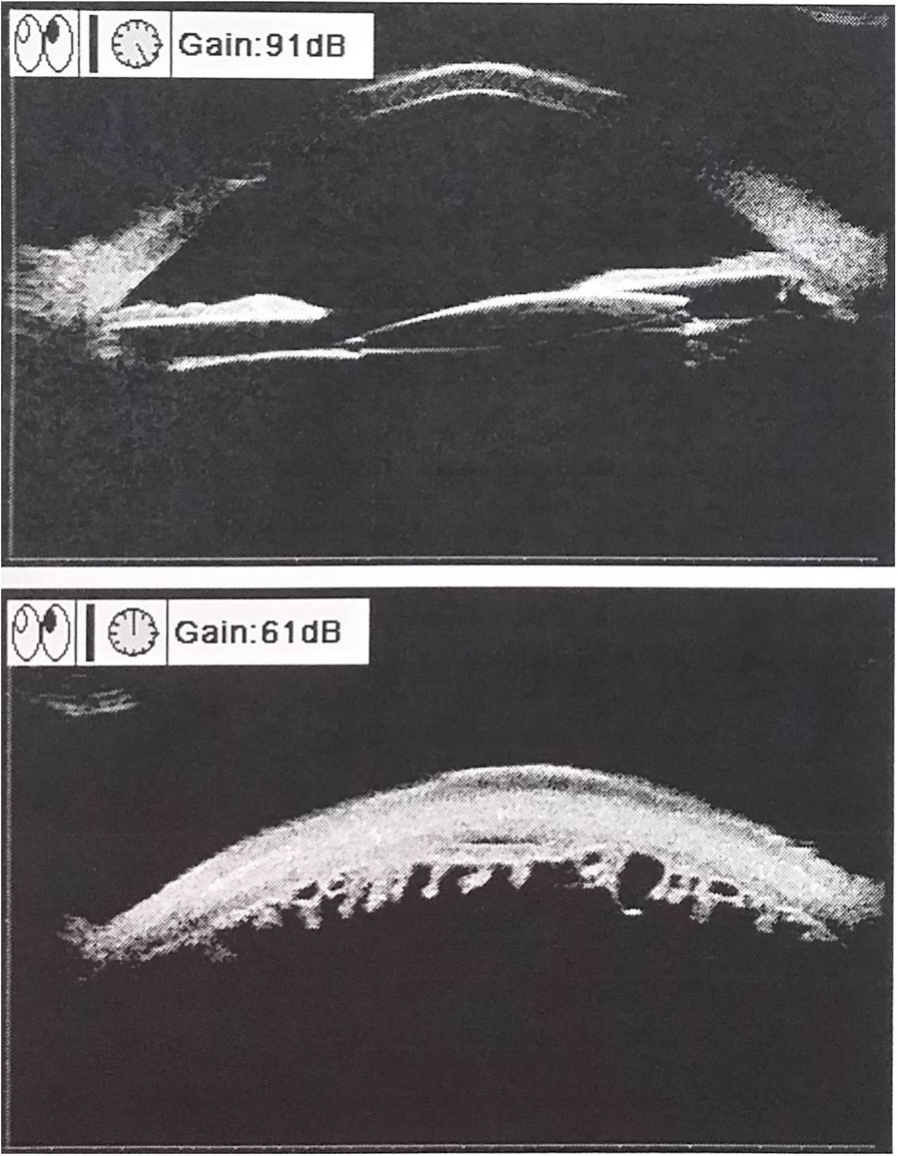
Ultrasonic biomicroscopy image of the left eye. This image shows moderate intraocular lens (IOL) decentration and tilt, without detectable intraocular foreign bodies (IOFBs) in the anterior chamber, iris stroma, ciliary body, and pars plana

**Fig. 7:**
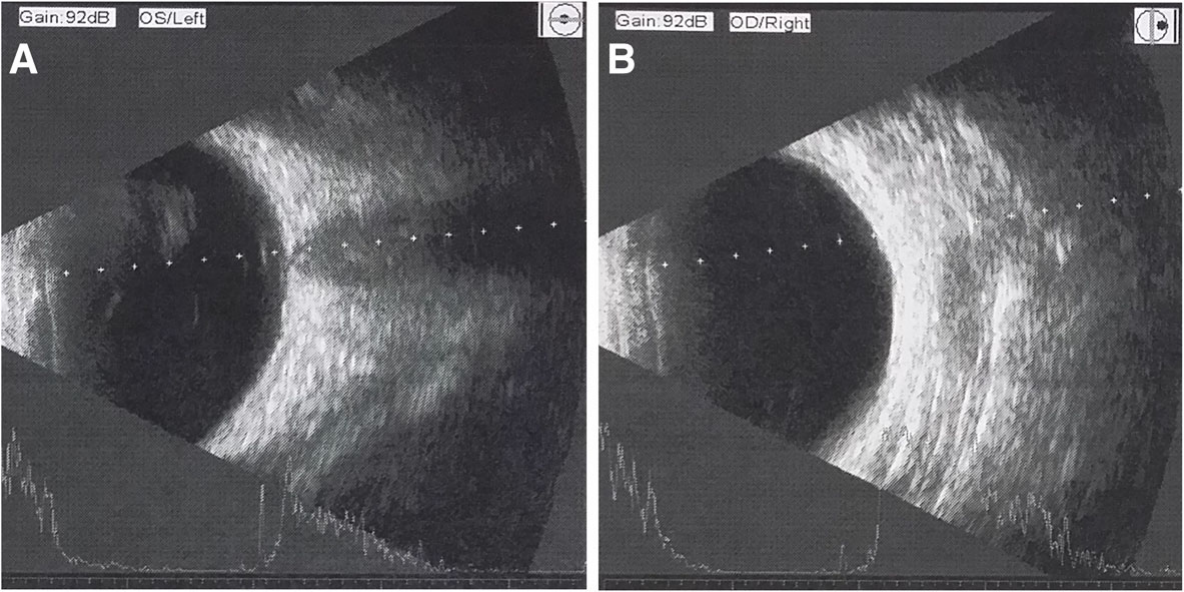
Ultrasound B-scan of the posterior segment in the left eye (**a**) and the fellow eye (**b**)

**Fig. 8:**
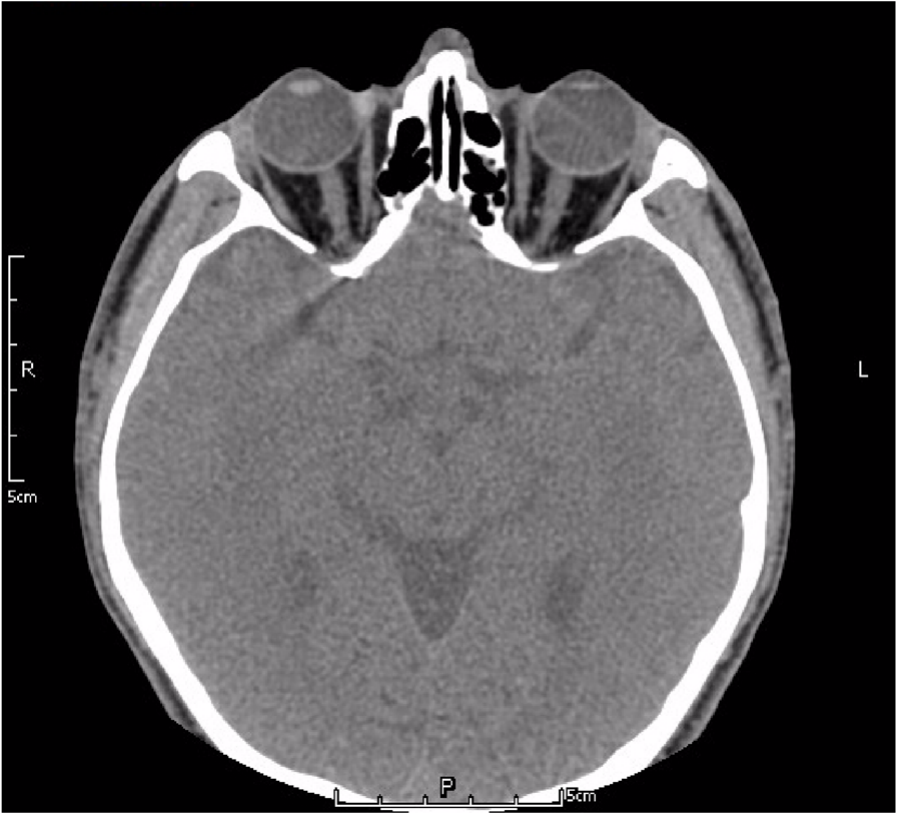
No intraocular foreign bodies (IOFBs) were identified on 0.625-mm thin-sliced helical CT

**Fig. 9:**
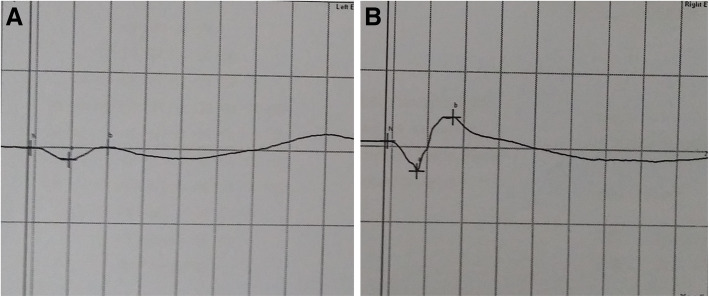
Electroretinography was normal in the right eye for scotopic response, while a remarkable amplitude loss was observed in the left eye

In the clinic, the visual acuity was 20/20 in his right eye (OD) and 4/20 in his left eye (OS). IOP was 13.2 mmHg OD and 34.5 mmHg OS, by Goldman applanation tonometry. Relative afferent pupillary defect (RAPD) OS was positive. Iris heterochromia and mild mydriasis OS was definite (Fig. 1). The left cornea was slightly thick and hazy with pigmented KP on the posterior surface without visible wounds or scars (Fig. 2). Gonioscopic examination uncovered a line of brownish deposits throughout the entire TM, and the anterior chamber angle was open without recession, synechiae or neovascularization (Fig. 3). Dilated fundus examination revealed retinal pigment epithelium (RPE) atrophy arranged in a leopard-spot pattern OS and obvious cup/disk asymmetry of 0.35 OD and 0.65 OS (Fig. 4). The three-mirror contact lens showed no retinal detachment or signs of IOFB. Spectral-domain optical coherence tomography (SD-OCT; RTVue-XR; Optovue, Inc., Fremont, CA) indicated central retinal thinning (Fig. 5). Using UBM (SW-3200, SUOER, China), no IOFB was detected in the anterior segment OS, including iris stroma and sclera (Fig. 6). An ultrasound B-scan (Aviso S, 10 MHz probe; Paris, France) showed moderately dense vitreous opacities with posterior vitreous detachment (PVD) (Fig. 7). The visual field, measured by Humphrey 30–2 perimetry (Zeiss Meditec, Dublin, CA, USA), showed a superior arcuate defect. An orbital X-ray and a 0.625-mm thin-sliced helical CT did not uncover any retained IOFB (Fig. 8). The amplitudes of the a and b waves were decreased on ERG (Roland Consult, Brandenburg, Germany) (Fig. 9).

**Fig. 10:**
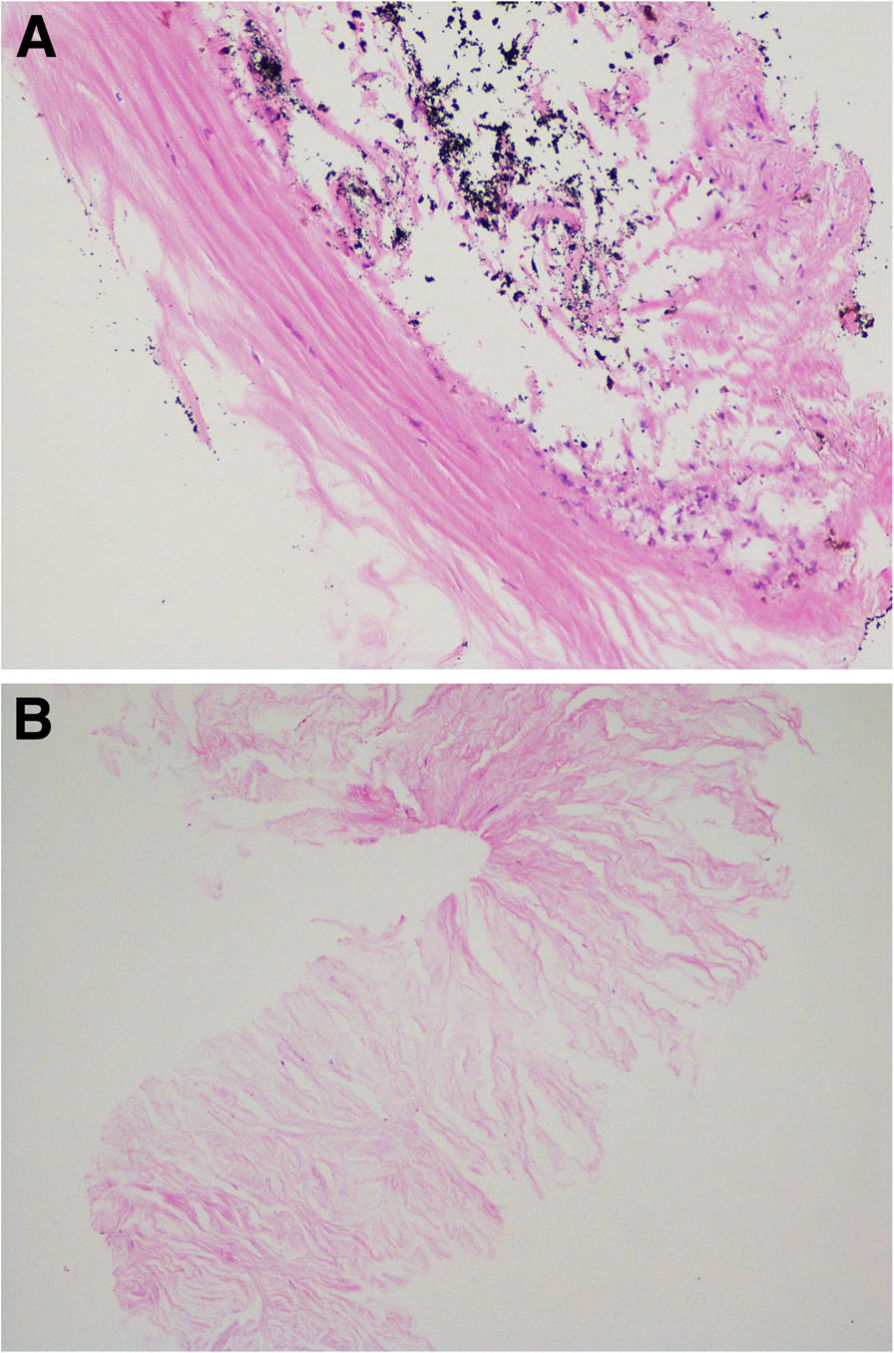
Histopathologic results for the trabecular meshwork (TM) with and without ocular siderosis. **a**, Prussian blue staining uncovered strong iron deposition in the TM of the left eye (black arrows). **b**, Iron pigments were not detected in the TM of a patient with primary open angle glaucoma (POAG)

In view of the above-mentioned findings and his occupation, although lacking evidence of radiologically detectable IOFB, the patient was still diagnosed with siderotic glaucoma. Carteolol 2% bid, brimonidine 0.2% tid and latanoprost 0.005% at bedtime were promptly initiated OS. IOP decreased to 21.3 mmHg within 3 days, and an uneventful trabeculectomy with mitomycin C (MMC) in his left eye was performed by an experienced surgeon (Z.H.L.). In brief, the surgical procedures were all performed under local anesthesia. After the fornix-based superior conjunctiva and Tenon’s capsule were dissected, a square-shaped superior scleral flap of 3 mm × 4 mm and 1/2 the thickness of the sclera was prepared. MMC (0.02%, 0.2 mg/mL) was placed under the scleral flap and between the sclera and Tenon’s capsule for 2 min with several sponges. The sponges were then removed and 40 mL balanced salt solution was used to irrigate the surgical area carefully. A paracentesis at 2 o’clock was created with a 15-degree knife, followed by a sclerotomy and a peripheral iridectomy with a Vannas scissor. The TM tissue obtained during surgery was stained with specific Prussian blue. The results verified obviously scattered iron deposits (Fig. 10a). Another sample was obtained from another patient diagnosed with primary open-angle glaucoma (POAG) as a control, and no obvious iron pigments were detected (Fig. 10b). One day after surgery, the IOP OS was 12.7 mmHg. The patient was followed-up for approximately 6 months. At the final examination, visual acuity was 5/20, IOP was 14.3 mmHg without medications and no progressive retinal abnormalities were detected.

## Discussion and conclusions

Our patient presented with a six-month history of progressive blurred vision, elevation of IOP, and a change in the colour of his left eye. Slit-lamp examination revealed characteristic iris heterochromia, mild mydriasis and brownish deposits throughout the TM. No obvious corneal macula was observed. Although no solid proof of an IOFB was verified on UBM, ultrasound B-scan, or 0.625-mm thin-sliced helical CT, the possibility of siderotic glaucoma resulting from the deposition of an iron-containing IOFB could not be excluded. With the aid of ERG and histopathology (Prussian blue staining) after trabeculectomy, the patient was eventually diagnosed with siderotic glaucoma.

As iron undergoes dissolution over time and due to the limitations of ocular imaging, IOFB is not always detectable in ocular siderosis or siderotic glaucoma [12]. CT scan, ultrasound B-scan, and UBM are vital adjunct imaging tools for IOFB management [13]. CT of the orbit without contrast is the recommended approach to detect orbital foreign bodies and IOFBs. Compared with conventional CT, thin-sliced helical CT is more sensitive [14]. However, if the foreign bodies are too small or if the eye moves, the IOFB may be missed. When an IOFB cannot be visualized directly or with CT scan, a real-time, high resolution ultrasound B-scan may be helpful [15]. Nevertheless, ultrasonography can disrupt the normal anatomy of the eye and requires sophisticated machinery and skilled operators [16]. High frequency UBM (50 MHz) is a valuable technique for detecting small and anteriorly located foreign bodies in the anterior chamber and iris stroma, around the ciliary body and ciliary processes, and within the retrolental space. In this case, 0.625-mm thin-sliced helical CT, high resolution ultrasound B-scan, and UBM were used, but no foreign body was observed. Thus, we postulated that IOFB dissolution may be one of the possible reasons for our inability to detect it radiologically.

Siderosis bulbi without a radiologically detectable foreign body is rare, and has been reported as a result of intracorneal, intralenticular, or intrascleral rust residue [12, 17, 18]. In this case, we ruled out the possibility of rust deposition in the cornea and sclera with slit-lamp and UBM examination. Considering that the middle-aged blacksmith was once diagnosed with traumatic cataract at a local hospital, an intralenticular rust residue was possible, and it may have been overlooked while the patient was undergoing cataract surgery.

The onset of blurred vision, iris heterochromia, and elevation of IOP, which characterize siderotic glaucoma, may also be seen with Fuchs’ heterochromic uveitis (FHU), creating a differential diagnostic problem. Heterochromia is always regarded as a hallmark of an FHU diagnosis, while it is often absent in heavily pigmented eyes. In contrast, a certain degree of iris atrophy may be obvious [19]. In our case, no distinct iris atrophy was found, but the heterochromia was remarkable. Moreover, similar to previous studies, a special Prussian blue staining in our patient showed scattered iron deposition in the TM [20]. Electrophysiology also revealed typical amplitude loss on ERG. Hence, a diagnosis of siderotic glaucoma seems to be rational.

In summary, this report underlines the importance of the close monitoring of patients with a history of high-velocity metallic ocular trauma, even if there is no definite radiologically detectable IOFB, as they could develop siderosis bulbi later. When decreased visual acuity, elevated IOP, iris heterochromia and brownish deposits on the anterior trabecular meshwork are identified in a patient suffering from ocular trauma, mitomycin C augmented trabeculectomy followed by histopathologic study is pivotal to clarify the diagnosis. In addition, ERG prior to surgery and during follow-up is valuable in the prognosis of siderosis bulbi.

## Data Availability

All the data supporting our finding is contained within the manuscript.

## Abbreviations

ERG: Electroretinography
FHU: Fuchs’ heterochromic uveitis
IOFB: Intraocular foreign body
IOL: Intraocular lens
KP: Keratic precipitates
OD: Right eye
OS: Left eye
RAPD: Relative afferent pupillary defect
UBM: Ultrasonic biomicroscopy
